# Recurrent Mobility: Urban Conduits for Diffusion of Energy Efficiency

**DOI:** 10.1038/s41598-019-56372-4

**Published:** 2019-12-27

**Authors:** Neda Mohammadi, John E. Taylor

**Affiliations:** School of Civil and Environmental Engineering, Georgia Institute of Technology, Atlanta, GA 30332-0355 United States

**Keywords:** Energy and society, Applied physics

## Abstract

Recent advances in energy technologies, policies, and practices have accelerated the global rate of improvements in energy efficiency, bringing the energy targets identified in the 2030 United Nations (UN) Sustainable Development Agenda within reach. However, Target 7.3 requires this rate to double by 2030, demanding a more substantial response to energy interventions. At present, energy interventions are failing to reach optimal levels of adoption in buildings, which are the largest urban energy consumers. This is due to a combination of direct and indirect effects generally referred to as the energy efficiency gap. Here, we compare over 18.8 million positional records of individuals against Greater London’s buildings energy consumption records over the course of one year. We demonstrate that indirect (i.e., spillover) effects, arising from *recurrent mobility*, govern the diffusion of urban buildings’ energy efficiency, far outpacing direct effects. This has been understood as a consequence of underlying spatiotemporal dependencies at the intersection of energy use and social interactions. We add to this the critical role of recurrent mobility (i.e., the mobility of those urban populations who repeatedly visit certain locations, such as home and work) as a diffusion conduit. These findings suggest that in order to improve the current levels of adoption, interventions must target times and locations that function as dense hubs of energy consumption and social interactions. Recurrent mobility thus provides a viable complement to existing targeted intervention approaches aimed at improving energy efficiency, supporting efforts to meet the UN’s 2030 energy targets.

## Introduction

New energy technologies, policies, and practices are being introduced in nations around the world in an attempt to meet the 2030 energy efficiency targets set by the United Nations (UN) Agenda for Sustainable Development, but despite substantial advances in energy efficiency under the UN’s Sustainable Development Goal (SDG) 7^1^, progress is uneven and slow^2,3^. The rate of energy efficiency improvements achieved so far falls well short of the 2.6% annual cut needed to reach Target 7.3, namely to double the global rate by 2030, due to underperformance in the years prior to the adoption of the agenda, necessitating a much more substantial response to energy interventions^4^. Among the various options, buildings, which are historically major consumers of energy in many regions of the world, continue to offer the greatest potential for improvements in energy efficiency^5^. In high-income, industrialized cities such as London, Tokyo, New York, Berlin, and Seoul, residential and commercial buildings are responsible for over 50% of the total energy consumption^6^. Interventions that are specifically targeted at promoting energy-efficient technologies and practices in buildings therefore represent an obvious first step towards addressing this issue.

However, despite extensive research indicating the promise of social benefits, financial mitigations, and environmental gains, many such interventions are only slowly starting to being adopted by consumers and businesses, seldom achieving justifiable levels of private (that would payoff for the adopter) and social (that would have social benefits) gains^7^. This phenomenon, referred to as the energy efficiency gap, is primarily characterized by a range of barriers hampering the delivery of energy efficiency, most of which can be described as either market or behavioral failures^7,8^; energy efficiency interventions thus generally target either *information* or *behavior* problems. Information problems^9,10^ are a form of market failure identified as externalities with a relatively large impact on the social energy efficiency gap. They include imperfect^9^ or asymmetric information^8^ that can cause systematic underinvestment and undermine the effectiveness of certain building policies^8^. Behavioral problems are internalities that lead to a private energy efficiency gap and are rooted in social norms^11^ and other forms of social influence, such as personalized energy use feedback^12^, social comparison^13^, and prosocial behavior incentives like reputation or self-respect^14^, all of which can cause systematic biases in consumer decisions^8^. Interestingly, despite their potential to significantly improve the rate at which the energy efficiency gap can be closed^15^, current energy efficiency interventions are heavily focused on targeting these problems in an isolated way, investigating them solely from a cause-and-effect and economic rationality perspective. This means that they tend to overlook the spatiotemporal conditions within which interventions can most effectively take place to exploit opportunities for large scale consumer responses. Even though changes at large social and temporal scales have the potential to generate the greatest results in closing the energy efficiency gap^15^, there remains a general lack of scientific understanding of the social, spatial, and temporal dimensions at which interventions can most effectively reach specific target populations. In order to improve the effectiveness of targeted interventions, it is thus important to not only correctly identify, but also to reach the target populations more effectively if we are to encourage participation by those most likely to achieve the greatest energy savings. This implies not only including the target populations, but also excluding the non-target populations from well-crafted energy efficiency interventions. This is a serious omission: determining the link between the distribution of building energy consumption and social interactions in time and space is a critical step in identifying the most effective time- and place-specific targets for energy efficiency interventions at larger urban scales. Establishing spatiotemporally targeted interventions at a city-wide scale can increase the probability of intervention adoption by individuals and communities, boosting energy efficiency considerably.

Energy consumption patterns depend on daily human activities^16^, with certain types of energy use behavior creating clusters in specific spatial and temporal locations^17^. These can include work, home and leisure activities, all of which have an impact on the energy saving potential in distinct areas of the city. Research quantifying the underlying spatiotemporal structure for urban energy consumption has confirmed the presence of direct and indirect (i.e., spillover) effects among neighboring units^18^. These effects, which very often emerge from daily human activities, can shed light on the reasons for overestimates of the impact of the least-effective interventions, as well as underestimates of the impact of the most-effective interventions^19,20^, explaining when and where the energy efficiency gap most frequently manifests. It can also predict when and where undesired outcomes such as rebound effects^21^ may emerge due to complacency, as well as peer-effects that may either encourage pro-environmental behaviour^22^ or expedite the adoption of energy efficient technology^23^. In spite of this, measuring daily human activities at the city scale is non-trivial. Research has shown that one reliable proxy for daily human activities^24^ that can further provide insights on fluctuations of energy consumption^18^ is tracing people’s movements around the city^25^. Human mobility has been linked to social ties^26,27^, social behaviour^28^, influence^29^, knowledge flow spillovers^30,31^, patterns of diffusion in spread of infectious diseases^32,33^, and human sentiment^34^. It is dominated by a relatively small number of most frequently visited locations, or “primary habitats”^35^, and can thus reliably describe diffusive phenomena such as the widespread adoption of energy efficient technologies and practices via recurrent social encounters^36^ in urban environments. When attempting to target the consumers who will be most responsive to a particular intervention^8^, however, it is critical to understand whether the underlying spatial dependencies between energy consumption and social interactions are linked to the mobility of urban populations. In this study, we therefore sought to investigate the intra-city interdependencies that may exist between human mobility and energy consumption, and, to determine whether the distribution of urban energy consumption can be explained by patterns of individual human mobility. Our primary focus is on examining recurrent mobility, which is the human mobility of those who repeatedly visit certain locations (i.e., returners^37^). The returners population, namely those individuals whose mobility network is dominated by a few recurrent preferred locations (e.g., home, work), is known to exhibit significant correlations between their mobility networks and their individual social interactions, as well as their role in the diffusion phenomenon^37^. To unveil recurrent human interactions in space and over time that are indicative of social ties^36^, we classify individuals into returner populations with distinct mobility patterns^37^ and examine the degree to which the mobility, and by extension the daily human activity patterns and social interactions, of this population is associated with energy use^38,39^. Further, we explore the direct and indirect (i.e., spillover) effects of recurrent mobility—as an indicator for recurrent time- and place-based interactions of the urban population with local building energy systems—to identify the spatiotemporal capacities that will be most susceptible to energy efficiency interventions, enabling us to assess the potential utility of recurrent mobility as a conduit for the diffusion of energy efficiency measures.

## Interdependence of Human Mobility and Energy Consumption

**Figure 1:**
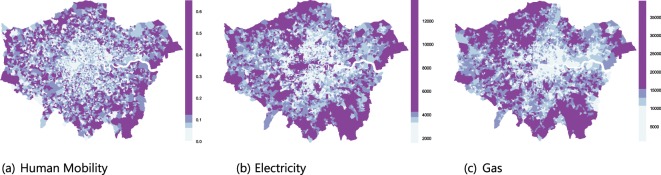
Spatial distribution of (**a**) human mobility (km), (**b**) residential electricity (kWh), and (**c**) gas (kWh) consumption in Greater London, LSOA-level, February 2014.

**Figure 2:**
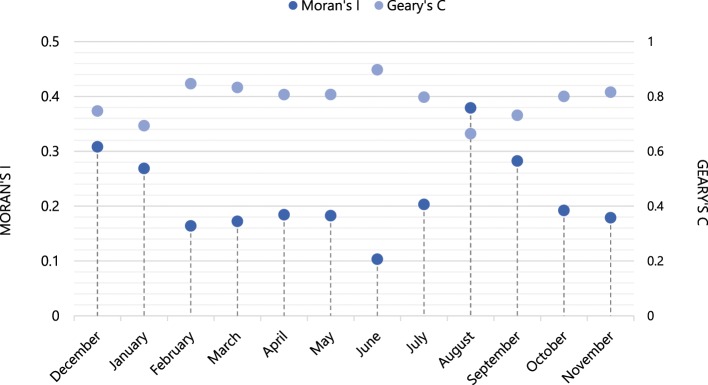
Spatial autocorrelation: Moran’s *I*, and Geary’s *C* of Human Mobility, Greater London 2014.

**Figure 3:**
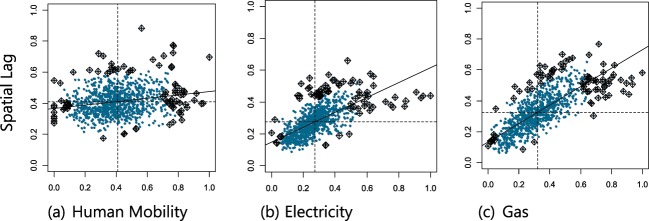
Moran Scatterplot for (**a**) human mobility, residential (**b**) electricity, and (**c**) gas consumption in Greater London, LSOA-level, February 2014.

In order to examine the energy consumption attributable to individuals’ urban mobility, we began by examining how human mobility and energy consumption are spatially distributed (Fig. 1). This included assessing whether there are underlying processes that impose structure on these distributions that can be used to quantify these patterns or whether they are merely characterized by spatial heterogeneity and randomness. We found that the spatial distribution of human mobility at the neighborhood level is not random; a unique underlying spatial structure governs the mobility of urban population across neighborhoods that are clustered together. This structure was present throughout the year with only insignificant deviations from the mean (Fig. 2). We thus rejected the null hypothesis of spatial randomness in favor of structure (i.e., spatial autocorrelation, *see Methods*), meaning that the spatial fluctuations of human mobility are relevant and provide additional insights into the structure. Observations of human mobility at one location correlate with those for neighboring locations, with a possible effect on the neighboring values (i.e., values for one spatial unit depend on the values at other neighboring locations). The presence of spatial structure suggests that the locations of the centers of mass of the individual mobilities (Eq. 1) will be significant, likely as a result of where and how individuals arrange their daily trips to home, work, school, shopping, leisure, and so on. Similar results were obtained for energy (electricity and gas) consumption. Figure 3 depicts the Moran scatterplots for energy consumption and human mobility in the month of February 2014 for Greater London. These spatial autocorrelations suggest predictive models that relate observations of human mobility or energy use at one location to those at other locations, and can be used to define their particular spatial correlation structure more effectively.

Once the existence of a spatial structure for both human mobility and energy consumption was confirmed we asked: Is it likely that people’s mobility (representing their daily activity patterns) is the cause of the spatial processes (diffusion, social interaction, etc.) driving particular energy use patterns in particular locations? If so, does our data support this? Given the spatial autocorrelations, we conducted a spatial regression analysis *(see Methods)* to visually and statistically explore this question and determine precisely how the strength of the association between human mobility and energy consumption varies by area. A spatial regression analysis between human mobility and energy consumption (electricity and gas) across Greater London’s 4,835 spatial divisions was performed to evaluate the contributions of human activities to energy use. The results revealed that the spatial distribution of the energy consumption (Supplementary Fig. 4) was not independent of human mobility, rather the spatial imprints of human mobility could be used to localize the distribution of energy consumption, confirming the existence of statistically significant relationships between the two (Tables 1 and 2). These spatial dependencies were intermittent across the year, and the monthly difference was almost unnoticeable, reinforcing the finding of an underlying spatial structure between energy consumption and human mobility patterns (Supplementary Figs. 5 and 6).

**Table 1:** Human mobility-driven spatial regression analysis of electricity consumption, Greater London 2014.

Models	Winter			Spring			Summer			Fall		
	*December*	*January*	*February*	*March*	*April*	*May*	*June*	*July*	*August*	*September*	*October*	*November*
*Spatial Lag Model (SAR)*												
*p*-value	<2.2e-16	<2.2e-16	<2.2e-16	<2.2e-16	<2.2e-16	<2.2e-16	<2.2e-16	<2.2e-16	<2.2e-16	<2e-16	<2e-16	<2.2e-16
AIC^a^	76951 (78825)	76953 (78840)	76950 (78801)	76944 (78788)	76951 (78797)	76951 (78801)	76951 (78821)	76950 (78800)	76953 (78834)	76949 (78831)	76944 (78789)	76950 (78803)
Statistic	54.269***	54.499***	53.874***	53.67***	53.825***	53.851***	54.18***	53.807***	54.455***	54.391***	53.669***	53.796***
BP^b^	1.8873	3.9916	4.2984	4.3728	4.6953	2.3556	1.3981	2.8142	1.0499	3.186	2.6317	1.4847
Moran’s *I*^c^	−1.66e-02	−1.66e-02	−1.63e-02	−1.64e-02	−1.65e-02	−0.01654	−1.66e-02	−1.64e-02	−1.66e-02	−1.67e-02	−0.01598	−1.63e-02
*Spatial Error Model (SEM)*												
*p*-value	<2.2e-16	<2.2e-16	<2.2e-16	<2.2e-16	<2.2e-16	<2.2e-16	<2.2e-16	<2.2e-16	<2.2e-16	<2e-16	<2e-16	<2e-16
AIC^a^	77044 (78825)	77044 (78840)	77043 (78801)	77041 (78788)	77044 (78797)	77044 (78801)	77044 (78821)	77044 (78800)	77044 (78834)	77041 (78831)	77039 (78789)	77043 (78803)
Statistic	55.437***	55.538***	55.254***	55.019***	56.192***	55.486***	55.45***	43.878***	55.528***	55.415***	54.942***	55.25***
BP^b^	0.091264	0.60269	0.24841	0.75973	1.0731	0.13751	0.5995	0.10037	0.6214	0.91957	0.00061147	0.093808
Moran’s *I*^c^	−0.019201	−1.928e-02	−1.90e-02	−1.88e-02	−0.0192	−1.92e-02	−1.92e-02	−1.91e-02	−0.019298	−1.925e-02	−1.86e-02	−1.905e-02

*p* < 0.1*; *p* < 0.05**; *p* < 0.001***. ^a^AIC for ordinary least squares (OLS) models in parentheses. ^b^Breusch-pagan test for heteroscedasticity. ^c^Moran’s *I* for residuals.

**Table 2:** Human mobility-driven spatial regression analysis of gas consumption, Greater London 2014.

Models	Winter			Spring			Summer			Fall		
	*December*	*January*	*February*	*March*	*April*	*May*	*June*	*July*	*August*	*September*	*October*	*November*
*Spatial Lag Model (SAR)*												
*p*-value	<2.2e-16	<2.2e-16	<2.2e-16	<2.2e-16	<2.2e-16	<2.2e-16	<2.2e-16	<2.2e-16	<2.2e-16	<2.2e-16	<2.2e-16	<2.2e-16
AIC^a^	90944 (93886)	90944 (93895)	90936 (93788)	90929 (93797)	90941 (93796)	90939 (93797)	90946 (93893)	90936 (93786)	90946 (93905)	90946 (93917)	90933 (93795)	90937 (93812)
Statistic	75.038***	75.099***	73.437***	73.554***	73.466***	73.526***	75.282***	73.362***	75.253***	75.606***	73.562***	73.757***
BP^b^	0.2323	0.00028	10.951	4.7992	6.2904	6.1036	1.3455	4.0509	0.92644	1.2326	8.498	8.6573
Moran’s *I*^c^	−3.947e-02	−0.03918	−3.98e-02	−3.964e-02	−3.969e-02	−3.958e-02	−3.915e-02	−3.965e-02	−3.913e-02	−3.89e-02	−3.959e-02	−3.943e-02
*Spatial Error Model (SEM)*												
*p*-value	<2.2e-16	<2.2e-16	<2.2e-16	<2.2e-16	<2.2e-16	<2.2e-16	<2.2e-16	<2.2e-16	<2.2e-16	<2.2e-16	<2.2e-16	<2.2e-16
AIC^a^	90946 (93886)	90947 (93895)	90947 (93788)	90945 (93797)	90945 (93796)	90947 (93797)	90945 (93893)	90947 (93786)	90947 (93905)	90944 (93917)	90946 (93795)	90947 (93812)
Statistic	75.604***	76.151***	76.034***	74.98***	75.487***	75.549***	75.645***	75.546***	78.104***	75.641***	75.205***	76.657***
BP^b^	0.0030247	0.004476	2.1066	0.30835	0.979	0.12253	0.030219	0.046681	0.51677	0.57309	2.2661	2.7801
Moran’s *I*^c^	−3.910e-02	−3.907e-02	−3.906e-02	−3.877e-02	−3.942e-02	−3.917e-02	−3.928e-02	−3.908e-02	−0.039128	−3.909e-02	−0.03888	−3.902e-02

*p* < 0.1*; *p* < 0.05**; *p* < 0.001***. ^a^AIC for ordinary least squares (OLS) models in parentheses. ^b^Breusch-pagan test for heteroscedasticity. ^c^Moran’s *I* for residuals.

The results of the spatial regression analysis indicate that the strength of the association between human mobility and energy consumption depends on spatial location, which can further be contextualized more locally based on “primary habitats”. This means that human mobility across different areas in Greater London, an indicator of daily human activities including social interactions, can indeed be regarded as a proxy indicator for spatial fluctuations in energy consumption, with changes in human mobility explaining shifts in the pattern of energy consumption.

## Spillover Effects of Recurrent Mobility

Perhaps the most striking example of the effects originated from patterns of human mobility on urban energy consumption, and the significant contribution of these interdependencies, is the possible indirect spatial (spillover) effect^40^. Here, an indirect influence can span borders and diffuse to spatially adjacent areas when social interactions (e.g., social imitation and peer effects) take place across spatial borders. This effect determines whether fluctuations in energy use due to recurrent human mobility within a single spatial unit (an individual Lower Super Output Area (LSOA) in this context) exhibit any significant diffusive effects associated with the returner population. For example, while spatial dependencies (e.g., geographical similarities such as sun exposure and solar potential) among spatially adjacent regions contribute to the adoption of solar photovoltaic (PV) installations and the emergence of solar clusters^23,41^, research suggests that the adoption rate is also driven by spillover effects. These effects can range from the peer effects arising due to the visibility of solar panels to the public (e.g., a returner passerby notices recent solar PV installations in the area where he or she works), through more deliberate efforts towards adoption (e.g., Solar Community Organizations that promote the adoption of residential solar PV^42^), to other drivers of spillover effects in PV uptake, including the spatial dependence of knowledge and technology diffusion at different scales (e.g., neighborhoods, cities, states, and countries). We can conceptualize two underlying spatial structures in which the dynamics of the diffusion of energy consumption take place, namely energy consumption (electricity and gas) and recurrent mobility, and take into account the dependency of energy consumption on human mobility to determine the spatial reach of spillover effects. To investigate whether any diffusive effect such as the spatial spillover effect exists among the neighboring areas, we developed a spatial Durbin model (*see Methods*) and assessed the magnitude and significance of direct, indirect (i.e., spillover), and total effects. This model determines how changes in human mobility and energy use in a particular spatial unit will diffuse to all other neighboring units and hence how they are likely to, directly or indirectly, affect the energy use at those units.

**Figure 4:**
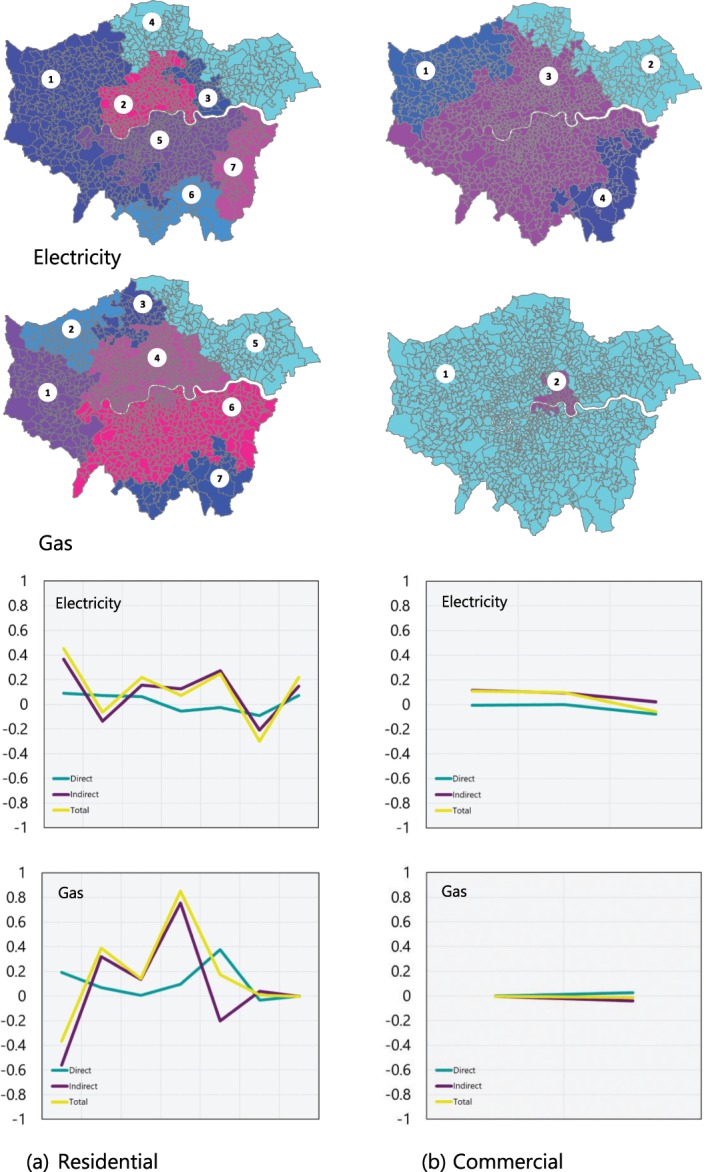
Direct and indirect (i.e., spillover) effects of recurrent mobility on energy consumption in (**a**) residential, and (**b**) commercial buildings, Greater London, May 2014. The y-axes indicate the effect estimate, and the x-axes represent the labeled spatial clusters.

Interestingly, the statistical significance of these effects imply that the effects of recurrent mobility and energy consumption predominantly exhibit an indirect (i.e., spillover) effect in both residential (Fig. 4(a)) and commercial (Fig. 4(b)) buildings and comprise the largest proportion of the total effects. The effect appears to be especially prominent for residential buildings, suggesting that recurrent mobility likely generates a stronger spillover in residential neighborhoods. Our findings indicate that, as a result of the underlying spatial dependencies that arise at the intersection of energy use and human mobility across spatially-constrained clusters of the corresponding neighborhoods, the spatial reach of spillover effects fluctuates across time and space (Supplementary Table 4). In smaller neighboring clusters, the spillover effect may dissipate quite rapidly and its spatial reach may approach zero after a comparatively short distance. However, as higher order neighbors are approached, the effect can decay more slowly. This suggests that there is a weaker direct effect on energy consumption compared to the exogenous effects from recurrent mobility that take place in its surroundings. The recurrent mobility driven effects found here indicate the broader perspective needed when devising targeted energy interventions over a larger scale.

## Discussion

Much of our current understanding of energy efficiency intervention programs for buildings is built on decades of research into ways to improve efficiency, boost cost-effectiveness, and explore the welfare implications. As a result, our energy interventions are often informed by similar insights primarily targeting consumer decisions^8^ in an isolated way solely from a cause-and-effect perspective. Despite the importance of the role time and place of energy use plays in the transformation of energy efficiency in cities, reflections on the spatiotemporal distribution of energy consumption in relation to daily human activities and social interactions are largely absent in energy intervention studies. Human mobility in urban areas, which is indubitably linked to daily human activity patterns and social interactions^37^, and its impact on the spatial distribution and spillover effects of energy consumption^38^, can thus serve as a quantitative representation of how energy efficiency interventions can be best targeted in space and over time. Indeed, characterising human mobility as a possible indicator for the induced fluctuations in the spatial distribution of energy consumption may even help explain why cities with higher mobility flows are considered influential places of culture and information^29^.

The findings of this study extend our understanding of the spillover effects of energy use beyond merely spatial proximity by quantifying a new measure for this effect. The consistent patterns we identified in the spatial dependencies of human mobility and energy use of the population over the course of the year reveal a predictable spatiotemporal pattern within various urban spatial units. Spatial dependence is the product of underlying location-specific activity processes that lead to clusters of mobility patterns and energy use. These patterns can potentially be explained by socioeconomic factors in spatial units that drive similar behavior, and/or externalities and spatial spillovers with fluctuating spatial reach^40^, enabling us to identify the interdependencies between energy consumption, specific urban spatiotemporal features, and individual activities. These include, for example, spatial interactions^43^, where individuals tend to interact with those who are spatially closer to them, and dispersal processes, where individuals travel short distances (e.g., home to work) and transfer their knowledge and energy use patterns with them. Accurate information on the spatial interdependence between fluctuating patterns of human mobility and energy use, can help define a predictable structure for targeting interventions.

Similarly, recurrent mobility can be indicative of the repeating co-located individual activities and social interactions of an urban population, which concurrently account for hotspots of energy consumption. Recurrent mobility patterns can reveal important information about the way citizens interact with their surroundings, hence driving energy use. This applies to groupings of populations with similar recurrent activity patterns and daily routines^37^, or diffusion processes^43^, where individuals in the same spatial divisions influence, acquire information, and adopt specific energy use patterns. The effects arising from recurrent individual activities and social interactions, whether direct or indirect (i.e., spillover), can both positively and negatively impact the waves of energy efficiency information and influence diffusion. The significant indirect effects found in our study for both energy consumption and recurrent mobility clusters confirm that the attributes of surrounding locations, as well as the individual activities and social interactions, are important determinants of energy use. Extensive concentrations of recurrent mobility shape spillovers as a uniquely effective phenomenon in cities^44^, to the extent that they can become the city’s “engines of growth”^45^ that target the most responsive consumers^8^.

Providing a clear picture of the diverse nature of the spatial reach of energy spillovers and its drivers at an urban scale, our findings establish a useful foundation for the development of localized and contextualized intervention strategies to mitigate the energy efficiency gap as it relates to the *information* and *behavior* barriers hindering the adoption of energy efficiency technologies and practices. In particular, they provide valuable information on how individual targeting strategies can be scaled up most effectively by incorporating patterns of recurrent mobility to achieve a larger return on investment and contribute to closing the energy efficiency gap. Knowing the social, spatial, and temporal conditions that appear to have the strongest influence on these spillover effects can facilitate larger scale targeted interventions that reach the target and eliminate the non-target populations. This includes both providing *information* at the point of decision making and targeting *behaviors* in a particular population^15^ (e.g., returners) whose participation potentially has the greatest impact. One significant implication of this new approach is that it becomes possible to determine how changes in energy use within each spatial division will be diffused across neighboring locations and, consequently, help predict how the extent of this diffusion is likely to fluctuate across different spatial divisions. The availability of such information will allow planners, city managers, and policy makers to identify hotspots within decentralized areas and develop effective interventions to influence energy use at the corresponding locations by either creating more substantial positive energy efficient spillover effects with greater spatial reach, or restricting undesirable or excessive energy use spillover effects, both of which may lead to more effective energy efficiency opportunities^46^. Future research should therefore explore such effects and their corresponding magnitudes in relation to changes in recurrent mobility. When creating such strategies, individual energy consumption hotspots can be targeted based on the spatial attributes of those locations. Diffusing the desired effects by introducing changes in the spatial structure by targeting specific buildings or areas to enhance the spillover effects, or instigating contagion by introducing changes in the flow by targeting specific clusters of population, will bring urban planners a step closer to achieving better management and allocation of scarce energy resources in cities.

Cities, which are dense energy-intensive hubs of human activities, are expected to accommodate nearly 70 percent of the world’s population by 2050^47^, creating the largest concentrations of activity-based energy consumption in human history with a superlinear growth curve^48^. Achieving global sustainable development is thus highly dependent on the success of cities in transforming towards sustainability^49,50^. Without understanding where and when targeted interventions will be most influential, efforts to accelerate intervention adoption and improve energy efficiency may dissipate, and be ineffectual. Identifying spatial regions with similar temporal activities should allow us to more accurately assess their likely energy use changes and thus optimize the distribution of energy provision. Treating the returners population as “agents of change”^51^, researchers, policy makers, and practitioners may be able to create positive spillover effects in and across cities’ primary habitats. Recurrent mobility patterns can thus become the focus of attention and a conduit for diffusion of energy saving practices and targeted energy efficiency interventions. Recurrently incorporating the human mobility-based fluctuations of energy consumption that occur due to shifting urban population dynamics may enable interventions to be directed at the most impactful areas of the city. Energy and other city infrastructure systems are critical in achieving urban sustainable development^52,53^. This will increase cities’ potential for reducing waste in the financial resources municipalities invest in energy efficient technology and infrastructure, and contribute to closing their energy efficiency gap, supporting efforts to meet the SDG 7 targets and thus the 2030 UN sustainable development agenda.

## Methods

### Datasets

The primary datasets used in this study consist of the energy consumption (both electricity and gas) and positional records of individuals across multiple spatial divisions (i.e, statistical areas) in Greater London during the year 2014. Social media data is known to have unique advantages for urban-sustainability research^54^. Geocoded digital records of social media activity broadly serve as a proxy for measures of individual human mobility^29,34,55,56^; this data, an alternative to call detail records (CDRs)^28,57^, is often preferred due to its ability to capture worldwide global behaviors compared to more locally accessible mobile phone activity records. Examining human mobility patterns of individuals in this study, we used 18.8 million individual positional records from Twitter, one of the largest social media networks in the world. Each public Twitter digital record (Tweet) contains an anonymized author ID, a unique post ID, the text of the post, a timestamp showing when it was posted, and the poster’s geolocation, where voluntarily publicly shared by the author. In order to account for demographic effects such as the varying tendencies of different segments of the urban population to use online social networks, as well as the authenticity of the data associated with specific individuals, we have followed Ruths and Pfeffer’s^58^ suggested data collection and methodology strategies. This includes, for example, applying filters that require a minimum number of data points from each individual to eliminate non-human accounts; account for platform and proxy population biases by including results for cities in different countries; and account for platform-specific algorithms by showing results for time-separated datasets from the same platform over multiple seasons. We also examined the demographics of Twitter users in Greater London^59^ (Supplementary Table 2), and concluded that our results are indeed representative of this population. Data from 3.4 million electricity meters and 3.0 million gas meters provided by the UK Department for Business, Energy & Industrial Strategy (www.gov.uk), were used as measures of electricity and gas consumption, respectively. The number of electricity and gas meters measured per statistical area are shown in Supplementary Table 1. The geographic information system (GIS) data includes the 2011 Census Geography Boundary Files in the Shapefile format provided by the UK Office for National Statistics (ONS) (www.ons.gov.uk), and the Greater London Authority (www.london.gov.uk). These statistical areas (Supplementary Fig. 2), generally referred to as super output areas (SOAs), expand three nested spatial scales: (1) 33 Boroughs; (2) 983 MSOAs (Middle Layer Super Output Areas); and (3) 4,835 LSOAs (Lower Layer Super Output Areas)^60^. LSOAs have a minimum size of 1,000 residents or 400 households and have an average of 1,500 residents; MSOAs have a minimum size of 5,000 residents or 2,000 households and have an average population size of 7,200 residents. Individual positional records base observations are processed and used to calculate the radius of gyration to capture individuals’ characteristic distance for their intra-urban mobility at various levels. The recurrent mobility datasets are then allocated to the appropriate MSOA- and LSOA-level statistical areas. Energy consumption record base observations (for both electricity and gas) are compiled using a bottom-up approach from an initial set of individual Meter Point Reference Numbers (MPRN), including the number of meters for both domestic and non-domestic (commercial and industrial) consumers. A difference between the two datasets, however, is that gas data are weather corrected (taking into account regional temperatures and wind speeds and incorporating trends), while the electricity data are not. These observation datasets are then aggregated at both a local and regional level utilizing postcode information and then allocated to the appropriate MSOA- and LSOA-level statistical areas.

### Quantifying recurrent mobility

We measure radius of gyration as the characteristic distance traveled by an individual *i* when observed up to time *t*^57^ and deviation of this measure from its corresponding center point *r*_*cm*_ (Eqs. 1 and 3) across different statistical areas *a*. Radius of gyration *r*_*g*_ (Eqs. 2 and 4), calculated at two spatial and two temporal levels (Supplementary Fig. 1), was used as an indicator for human mobility, which was selected from among the three most widely accepted indicators used to describe large-scale human mobility patterns— the radius of gyration ${r}_{g}(t)$, the trip distance distribution $p(r)$, and the number of visited locations $S(t)$^38,57,61^. Of these, the radius of gyration was deemed the most appropriate for capturing individuals’ characteristic travel distance within the areas where they habitually carry out their daily activities.

1 $${r}_{cmi}(t)=\frac{1}{{N}_{(t)}}\,\mathop{\sum }\limits_{i=1}^{{N}_{(t)}}\,{r}_{i}$$

2 $${r}_{gi}(t)=\sqrt{\frac{1}{{N}_{(t)}}\,\mathop{\sum }\limits_{i=1}^{{N}_{(t)}}\,{({r}_{i}-{r}_{cmi})}^{2}}$$

3 $${r}_{cma}(t)=\frac{1}{{K}_{(t)}}\,\mathop{\sum }\limits_{i=1}^{{K}_{(t)}}\,{r}_{cmi}$$

4 $${r}_{ga}(t)=\sqrt{\frac{1}{{K}_{(t)}}\,\mathop{\sum }\limits_{i=1}^{{K}_{(t)}}\,(\frac{{r}_{gi}(t)}{{r}_{gi}{(t)}_{{\max }}}).{({r}_{cmi}-{r}_{cma})}^{2}}$$

Here, *N* equals the total number of positional records per individual; and *K* equals the total number of individuals for whom a minimum 3 (three) positional records is available for a selected month during the study period (2014).

Recurrent mobility is measured by identifying the mobility of the returners^37^ population. We compared the total ${r}_{g}(t)$ and ${r}_{g}^{(s)}(t)$ of each individual through a bisector classification such that the population was split into two distinct classes: returners and explorers^37^. Returners, with ${r}_{g}^{(s)}(t)\approx {r}_{g}(t)$, are those individuals whose characteristic traveled distance is dominated by their *s*-th most frequently visited statistical area (e.g., MSOAs, as determined by Mohammadi and Taylor^38^), while the mobility network of *s*-explorers, with ${r}_{g}^{(s)}(t)\ll {r}_{g}(t)$, spanned multiple statistical areas and could not be reduced to *s* locations.

5 $${r}_{gi}^{(s)}(t)=\sqrt{\frac{1}{{N}_{s}}\,\mathop{\sum }\limits_{i=1}^{{N}_{s}}\,{({r}_{i}-{r}_{cmi}^{(s)})}^{2}}$$

*s* = 1, 2.

### Spatial autocorrelation

Spatial autocorrelation^62^ was used to assess the extent to which the spatial distribution of the data is compatible with spatial randomness and thus determine whether human mobility and energy consumption do indeed have spatial imprints. Spatial autocorrelation was used to test the spatial independence of human mobility and energy consumption across 4,835 LSOA statistical areas in Greater London. Moran’s *I*^63^ (Eq. 6), which ranges from −1 (most dispersed) to 1 (most clustered), was used to describe the degree of spatial concentration or dispersion for these variables, as originally examined by Mohammadi and Taylor^18^, with large values for *I* showing clusters of large values that are surrounded by other large values, namely (*I*+)– spatial clustering, and (*I*−)– spatial dispersion, indicating large values that are spatially enclosed by smaller values. While Moran’s *I* represents the global spatial autocorrelation for the data, Geary’s *C* ^64^ (Eq. 7) was also used based on the deviations in the responses of each observation with one another, ranging from 0 (maximum positive autocorrelation) to 2 (maximum negative autocorrelation), with 1 indicating an absence of correlation^38^. Moran’s *I* here serves as a measure of sensitivity to extreme values, with Geary’s *C* being used to evaluate the sensitivity to differences of energy consumption and human mobility values in smaller neighborhood LSOAs.

6 $$I=\frac{N\,{\sum }_{i=1}^{n}\,{\sum }_{j=1}^{n}\,{w}_{ij}({x}_{i}-\bar{x})\cdot ({x}_{j}-\bar{x})}{({\sum }_{i=1}^{n}\,{\sum }_{j=1}^{n}\,{w}_{ij})\cdot {\sum }_{i=1}^{n}\,{({x}_{i}-\bar{x})}^{2}}$$

7 $$C=\frac{(N-1)\,{\sum }_{i=1}^{n}\,{\sum }_{j=1}^{n}\,{w}_{ij}({x}_{i}-\bar{x})\cdot ({x}_{j}-\bar{x})}{2({\sum }_{i=1}^{n}\,{\sum }_{j=1}^{n}\,{w}_{ij})\cdot {\sum }_{i=1}^{n}\,{({x}_{i}-\bar{x})}^{2}}$$

Here, *n* represents number of observations on variable $\bar{x}$ at locations *i*, *j*, and *w*_*ij*_ are the elements of the weight matrix.

Spatial randomness is undesirable, so to ensure that it is not in effect, we reject the situation of spatial randomness in favor of structure (i.e., spatial autocorrelation). Spatial autocorrelation analysis exactly quantifies this (Supplementary Table 3), providing a measure of uncertainty (*p*-value) by which we can reject the null hypothesis (i.e., spatial randomness). A positive spatial autocorrelation indicates that similar values are clusters in neighboring locations, which would be a structure compatible with diffusion^65^.

### Spatial regression

In view of the spatial autocorrelation for human mobility and energy consumption, we investigated the nature of this structure through spatial regression models^66^ to examine the relationships between variables and their neighboring values and investigate the impact that one observation has on other proximate observations. Starting with an ordinary least squares model (Eq. 8), with the null hypothesis of a linear regression governing the structure of energy consumption by human mobility as a covariance.

8 $$y=X\beta +u$$

The expression describes the relationship between a vector of observations on the dependent variable *y (i.e., energy consumption)*, a matrix of observations on the explanatory variable *X* (i.e., human mobility), a vector of regression coefficients *β*, and a vector of error terms *u*. The error term is required to have constant variance and must be uncorrelated (i.e., to possess homoscedasticity)^38^.

While correlations explore the relationships between or among different variables, autocorrelations can be regarded as a special case, as they explore correlations within variables across space^67^. In the search for an appropriate autocorrelation structure for the data, we tested for deviations that would violate the null hypothesis such as a non-constant variance for error terms (i.e., heteroscedasticity), correlations for the error terms induced by Spatial Lag (SAR) (Eq. 9), or Spatial Error (SEM) models (Eq. 10)^38^. For the two mixed regressive models (simultaneous spatial autoregressive models consisting of both lag (SAR) and error (SEM) models), the SAR models with the lowest Akaike information criterion (AIC) predominantly provided the best representations of the global dependency conditions, expressed as

9 $$y=\rho Wy+X\beta +\varepsilon $$

where *y* is the dependent variable (i.e., energy consumption: electricity and gas); *X* is the regressive, independent (explanatory) variable (i.e., human mobility); *β* is the regression coefficient; $\varepsilon$ is a vector of random error terms; and $\rho$ is the spatial autoregressive coefficient in the spatial lag term $\rho Wy$, in which *Wy* represents the spatially lagged dependent variable *y*. Similarly, the SEM models are expressed as

10 $$y=X\beta +\lambda W\xi +\varepsilon $$

where *y* is the dependent variable (i.e., energy consumption: electricity and gas); *X* is the independent (explanatory) variable (i.e., human mobility); *β* is the regression coefficient; $\varepsilon$ is a vector of random error term; *λ* is the spatial autoregressive coefficient and $\xi$ represents the normal distribution $(0,\sigma 2I)$ in the term $\lambda W\xi$, in which $W\xi$ represents the spatial lag for the errors.

### Spatial spillover effect

The spillover effects are examined across the spatially constrained classes identified in Supplementary Fig. 2. First, the significance of an underlying spatial structure is examined through an exploratory spatial autoregressive analysis. We explore whether the spatial distribution of energy use in each clustering group is related to the urban infrastructure or human mobility attributes of its neighboring statistical areas and, if so, identify how they are associated and the extent of their direct, indirect (i.e., spillover), and total effects.

In order to partition the spatial divisions into spatial clusters that are reliable in terms of both attribute similarity and spatial similarity, we have adapted the graph-based SKATER algorithm^68^. The contiguity is taken into account by identifying the 983 MSOA statistical areas as nodes of an undirected weighted graph (Supplementary Fig. 3). Each MSOA is connected to its adjacent node if they share neighboring boundaries. Each connection has a value that represents the dissimilarity between the two nodes. A vector of ${x}_{i}=({x}_{ir},{x}_{ic},{x}_{im})$ consisting of numerical values for electricity and gas consumption, as well as human mobility, is associated with MSOA *i*. The weight associated with each connection measures the dissimilarities between MSOAs *i* and *j* with respect to their attribute vectors ${x}_{i}$ and ${x}_{j}$ as follows, representing the distance in multivariate space (Eq. 11):

11 $$d(i,j)=d({x}_{i},{x}_{j})=\mathop{\sum }\limits_{k=0}^{n}\,{({x}_{ik}-{x}_{jk})}^{2}$$

Higher values of the edge weights indicate that the corresponding MSOA pairs are farther apart in multivariate space. We first generated a minimum spanning tree (Supplementary Fig. 3) for the adjacency graph based on this pairwise distance measures of dissimilarities between MSOAs *i* and *j* by pruning the graph to minimize the sum of the intra-cluster square deviations. We then partitioned the spanning tree in order to maximize the internal homogeneity with respect to the energy use and mobility attributes, and minimize the overall dissimilarity to ensure that the clusters are internally the most similar.

The spatial interaction and spillover effects could then be measured via a Spatial Durbin Model^69,70^, in which the characteristics of a cluster are simultaneously considered in the analysis (Eq. 12). This model explicitly takes into account both the endogenous and exogenous interaction relationships, as identified by Mohammadi and Taylor.

12 $$y=\rho Wy+\alpha {I}_{n}+X\beta +WX\theta +\varepsilon \,\sim \,N(0,{\partial }^{2}{I}_{n})$$

where $y$ is an $n\times 1$ vector of energy use; $W$ is the spatial weight matrix, where $Wy$ represents the spatial lagged endogenous effects (e.g., urban infrastructure); and $\rho$ denotes the effect of y or the spatial autoregressive coefficient. ${I}_{n}$ is an $n\times 1$ vector of ones associated with the intercept parameter *α*. $X$ represents an $n\times 1$ matrix of human mobility measures related to the parameters $\beta$; $WX$ reflects the spatial lagged exogenous effects (e.g., human mobility); and *θ* denotes a $k\times 1$ vector of the effects of $WX$.

## Supplementary information

Supplementary Information

## Data Availability

The data that support the findings of this study are available from the public Twitter API (developer.twitter.com) but restrictions apply to their availability, which were used under license for the current study, and so are not publicly available. Data are however available from the authors upon reasonable request and with the permission of Twitter. The energy data can be accessed directly through the provider, the UK Department for Business, Energy & Industrial Strategy, and is available for both Electricity (www.gov.uk/government/collections/sub-national-electricity-consumption-data), and Gas (www.gov.uk/government/collections/sub-national-gas-consumption-data).

## References

[1] UN. SDGs. *Transforming Our World: The 2030 Agenda for Sustainable Development*. United Nations Department of Economic and Social Affairs (UN DESA), Available at, https://sustainabledevelopment.un.org/post2015/transformingourworld (2015).

[2] UN. Secretary-General. *Progress towards the Sustainable Development Goals: Report of the Secretary-General*, 18-07638. United Nations Economic and Social Council (ECOSOC), Available at, https://digitallibrary.un.org/record/1627573?ln=en (2018).

[3] Tracking progress on the SDGs. *Nat*. *Sustain*. **1**, 377–377, 10.1038/s41893-018-0131-z (2018).

[4] The World Bank. *Tracking SDG7: The Energy Progress Report*. International Bank for Reconstruction and Development, Available at, https://www.irena.org/publications/2018/May/Tracking-SDG7-The-Energy-Progress-Report (2018).

[5] IEA. *Energy Efficiency 2017.* International Energy Agency, 10.1787/9789264284234-en (2017).

[6] UN-HABITAT. *State of the World’s Cities 2008/2009: Harmonious Cities.* United Nations Human Settlements Programme (UN-HABITAT), Available at: http://mirror.unhabitat.org/categories.asp?catid=559 (2009).

[7] Gerarden TD, Newell RG, Stavins RN (2017). Assessing the energy-efficiency gap. J. Econ. Lit..

[8] Gillingham K, Keyes A, Palmer K (2018). Advances in evaluating energy efficiency policies and programs. Annu. Rev. Resour. Econ.

[9] Byrne DP, Nauze AL, Martin LA (2018). Tell me something I don’t already know: Informedness and the impact of information programs. Rev. Econ. Stat.

[10] Asensio OI, Delmas MA (2015). Nonprice incentives and energy conservation. Proc. Natl. Acad. Sci..

[11] Allcott H (2011). Social norms and energy conservation. J. Public Econ..

[12] Allcott H, Rogers T (2014). The short-run and long-run effects of behavioral interventions: Experimental evidence from energy conservation. Am. Econ. Rev..

[13] Jain RK, Gulbinas R, Taylor JE, Culligan PJ (2013). Can social influence drive energy savings? Detecting the impact of social influence on the energy consumption behavior of networked users exposed to normative eco-feedback. Energy Build..

[14] Bénabou R, Tirole J (2006). Incentives and prosocial behavior. Am. Econ. Rev..

[15] Stern PC (2016). Opportunities and insights for reducing fossil fuel consumption by households and organizations. Nat. Energy.

[16] Schipper L, Bartlett S, Hawk D, Vine E (1989). Linking life-styles and energy use: A matter of time. Annu. Rev. Energy Environ.

[17] Fonseca JA, Schlueter A (2015). Integrated model for characterization of spatiotemporal building energy consumption patterns in neighborhoods and city districts. Appl. Energy.

[18] Mohammadi N, Taylor JE (2017). Urban energy flux: Spatiotemporal fluctuations of building energy consumption and human mobility-driven prediction. Appl. Energy.

[19] Dietz T (2010). Narrowing the US energy efficiency gap. Proc. Natl. Acad. Sci..

[20] Attari SZ, DeKay ML, Davidson CI, Bruine de Bruin W (2010). Public perception of energy consumption and savings. Proc. Natl. Acad. Sci..

[21] Peschiera G, Taylor JE, Siegel JA (2010). Response–relapse patterns of building occupant electricity consumption following exposure to personal, contextualized and occupant peer network utilization data. Energy Build..

[22] Ayres I, Raseman S, Shih A (2013). Evidence from two large field experiments that peer comparison feedback can reduce residential energy usage. The J. Law, Econ. Organ..

[23] Bollinger B, Gillingham K (2012). Peer effects in the diffusion of solar photovoltaic panels. Mark. Sci..

[24] Sun L, Axhausen KW, Lee D-H, Huang X (2013). Understanding metropolitan patterns of daily encounters. Proc. Natl. Acad. Sci..

[25] Schneider CM, Belik V, Couronné T, Smoreda Z, González MC (2013). Unravelling daily human mobility motifs. J. Royal Soc. Interface.

[26] Lazer D (2009). Computational social science. Sci..

[27] De Domenico M, Lima A, Musolesi M (2013). Interdependence and predictability of human mobility and social interactions. Pervasive Mob. Comput..

[28] Toole JL, Herrera-Yaque C, Schneider CM, Gonzalez MC (2015). Coupling human mobility and social ties. J. The Royal Soc. Interface.

[29] Lenormand Maxime, Gonçalves Bruno, Tugores Antònia, Ramasco José J. (2015). Human diffusion and city influence. Journal of The Royal Society Interface.

[30] Oettl A, Agrawal A (2008). International labor mobility and knowledge flow externalities. J. Int. Bus. Stud..

[31] Filatotchev I, Liu X, Lu J, Wright M (2011). Knowledge spillovers through human mobility across national borders: Evidence from Zhongguancun Science Park in China. Res. Policy.

[32] Balcan D (2009). Multiscale mobility networks and the spatial spreading of infectious diseases. Proc. Natl. Acad. Sci..

[33] Wesolowski A (2012). Quantifying the impact of human mobility on malaria. Sci..

[34] Frank MR, Mitchell L, Dodds PS, Danforth CM (2013). Happiness and the patterns of life: A study of geolocated tweets. Sci. Reports.

[35] Bagrow JP, Lin Y-R (2012). Mesoscopic structure and social aspects of human mobility. PLoS One.

[36] Crandall DJ (2010). Inferring social ties from geographic coincidences. Proc. Natl. Acad. Sci..

[37] Pappalardo L (2015). Returners and explorers dichotomy in human mobility. Nat. Commun..

[38] Mohammadi N, Taylor JE (2017). Urban infrastructure-mobility energy flux. Energy.

[39] Mureddu, M., Facchini, A., Scala, A., Caldarelli, G. & Damiano, A. A Complex Network Approach for the Estimation of the Energy Demand of Electric Mobility. *Sci*. *Reports* **8**, 10.1038/s41598-017-17838-5 (2018). 10.1038/s41598-017-17838-5 PMC5762725 29321575

[40] LeSage, J. P. An introduction to spatial econometrics. *Revue D'économie Industrielle* **123,** 19-44, https://doi.org/10.4000%2Frei.3887 (2008).

[41] Rai V, Reeves DC, Margolis R (2016). Overcoming barriers and uncertainties in the adoption of residential solar PV. Renew. Energy.

[42] Mundaca L, Samahita M (2020). What drives home solar PV uptake? Subsidies, peer effects and visibility in Sweden. Energy Res. & Soc. Sci..

[43] Pan W, Ghoshal G, Krumme C, Cebrian M, Pentland A (2013). Urban characteristics attributable to density-driven tie formation. Nat. Commun..

[44] Glaeser EL, Kallal HD, Scheinkman JA, Shleifer A (1992). Growth in cities. J. Polit. Econ..

[45] Lucas RE (1988). On the mechanics of economic development. J. Monet. Econ..

[46] Armstrong RC (2016). The frontiers of energy. Nat. Energy.

[47] Kanyinda, A. *UN-Habitat Global Activities Report 2017*. United Nations Human Settlements Programme (UN-HABITAT), Available at: https://unhabitat.org/global-activities-report-2017 (2017).

[48] Bettencourt LMA, Lobo J, Helbing D, Kuhnert C, West GB (2007). Growth, innovation, scaling, and the pace of life in cities. Proc. Natl. Acad. Sci..

[49] Elmqvist T (2019). Sustainability and resilience for transformation in the urban century. Nat. Sustain..

[50] Acuto M, Parnell S, Seto KC (2018). Building a global urban science. Nat. Sustain..

[51] The role of society in energy transitions. *Nat*. *Clim*. *Chang*. **6**, 539–539, 10.1038/nclimate3051 (2016).

[52] Chester MV (2019). Sustainability and infrastructure challenges. Nat. Sustain..

[53] Thacker S (2019). Infrastructure for sustainable development. Nat. Sustain..

[54] Ilieva RT, McPhearson T (2018). Social-media data for urban sustainability. Nat. Sustain..

[55] Hawelka B (2014). Geo-located Twitter as proxy for global mobility patterns. Cartogr. Geogr. Inf. Sci..

[56] Wang Q, Phillips NE, Small ML, Sampson RJ (2018). Urban mobility and neighborhood isolation in America’s 50 largest cities. Proc. Natl. Acad. Sci..

[57] González MC, Hidalgo CA, Barabási A-L (2008). Understanding individual human mobility patterns. Nat..

[58] Ruths D, Pfeffer J (2014). Social media for large studies of behavior. Sci..

[59] Department for Culture Media and Sport (DCMS). *Taking Part Focus on: Social Media*. UK Statistics Authority, Available at: https://www.gov.uk/government/statistics/taking-part-april-2016-focus-on-reports (2016).

[60] ONS. *Statistical Bulletin: 2011 Census: Population and Household Estimates for England and Wales, March 2011.* Office for National Statistics, Available at: https://www.ons.gov.uk/peoplepopulationandcommunity/populationandmigration/populationestimates/bulletins/2011censuspopulationandhouseholdestimatesforsmallareasinenglandandwales/2012-11-23 (2012).

[61] Brockmann D, Hufnagel L, Geisel T (2006). The scaling laws of human travel. Nat..

[62] Fischer, M. M. & Wang, J. *Spatial Data Analysis: Models, Methods and Techniques* (Springer, Berlin, Heidelberg, 2011).

[63] Moran PAP (1950). Notes on continuous stochastic phenomena. Biom.

[64] Geary RC (1954). The contiguity ratio and statistical mapping. The Incorporated Stat.

[65] Anselin L (2003). Spatial externalities, spatial multipliers, and spatial econometrics. Int. Reg. Sci. Rev..

[66] Whittle P (1954). On stationary processes in the plane. Biom.

[67] Getis A (2007). Reflections on spatial autocorrelation. Reg. Sci. Urban Econ..

[68] Assunção RM, Neves MC, Câmara G, Da Costa Freitas C (2006). Efficient regionalization techniques for socio-economic geographical units using minimum spanning trees. Int. J. Geogr. Inf. Sci..

[69] Anselin L (1988). Spatial Econometrics: Methods and Models.

[70] Fischer Manfred M., Getis Arthur (2010). Handbook of Applied Spatial Analysis.

