# Supplementary material for: Recurrent Mobility: Urban Conduits for Diffusion of Energy Efficiency
Source: Sci Rep. 2019 Dec 27;9:20247. doi: 10.1038/s41598-019-56372-4 (PMC6934794; doi:10.1038/s41598-019-56372-4)
Supplement: Supplementary file 1 — Supplementary Information [file 41598_2019_56372_MOESM1_ESM.pdf]

*Supplementary Information for:*

# Recurrent Mobility: Urban Conduits for Diffusion of Energy Efficiency

**Neda Mohammadi and John E. Taylor**[*]

School of Civil and Environmental Engineering, Georgia Institute of Technology, Atlanta, GA 30332-0355
[*]jet@gatech.edu

## 1 Supplementary Figures

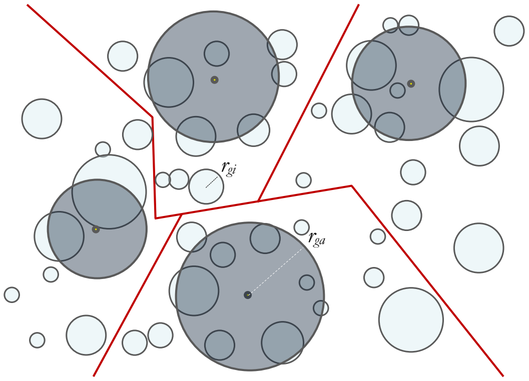


**Supplementary Figure 1.** Radius of gyration at the individual level $r_{gi}(t)$, and statistical area (e.g., LSOA) level $r_{ga}(t)$.

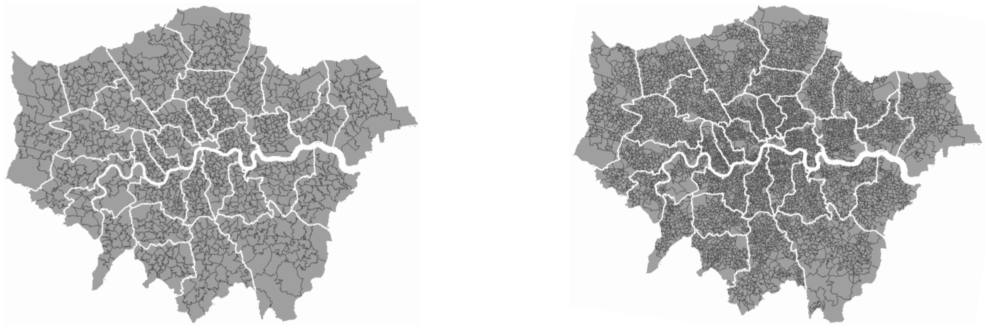

**(a)** BOR(33)-MSOA(983)

**(b)** BOR(33)-LSOA(4835)

**Supplementary Figure 2.** Nested statistical areas: BOR, MSOA, LSOA, Greater London (number of areas are given in parentheses).

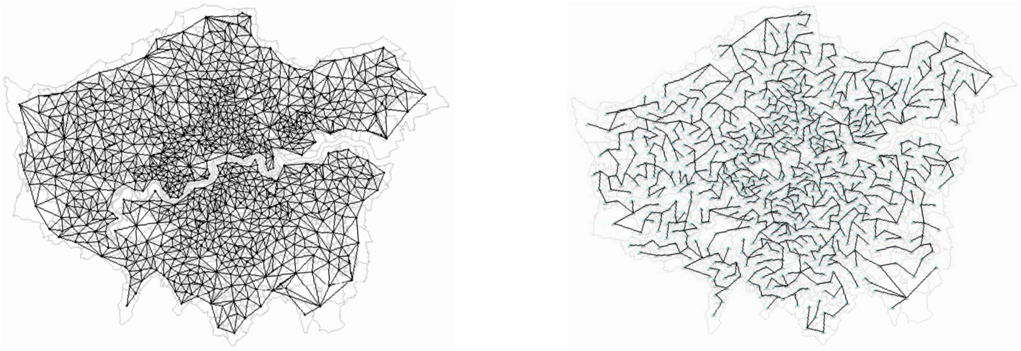

**(a)** Adjacency graph **(b)** Minimum spanning tree (MST)

**Supplementary Figure 3.** Spatially-constrained unsupervised clustering of MSOA-level statistical areas, Greater London.

# 2 Supplementary Tables

**Supplementary Table 1.** Number of **(a)** energy meters, and statistical areas **(b)** LSOAs, **(c)** MSOAs per Greater London Boroughs (BOR).

| | Greater London Borough | #Energy Meters | | #MSOAs | #LSOAs |
|---|---|---|---|---|---|
| | | *Electricity* | *Gas* | | |
| 1 | *Barking and Dagenham* | 73547 | 65715 | 23 | 110 |
| 2 | *Barnet* | 143079 | 126561 | 41 | 211 |
| 3 | *Bexley* | 96003 | 88773 | 29 | 146 |
| 4 | *Brent* | 113459 | 102149 | 34 | 173 |
| 5 | *Bromley* | 136999 | 128752 | 42 | 197 |
| 6 | *Camden* | 101209 | 79540 | 28 | 133 |
| 7 | *City of London* | 6321 | 2624 | 1 | 6 |
| 8 | *Croydon* | 149025 | 137219 | 45 | 220 |
| 9 | *Ealing* | 130831 | 117851 | 40 | 196 |
| 10 | *Enfield* | 124747 | 107151 | 37 | 183 |
| 11 | *Greenwich* | 107660 | 92355 | 38 | 151 |
| 12 | *Hackney* | 104160 | 91238 | 29 | 144 |
| 13 | *Hammersmith and Fulham* | 83025 | 74856 | 25 | 113 |
| 14 | *Harringey* | 105760 | 96417 | 37 | 145 |
| 15 | *Harrow* | 87799 | 81883 | 33 | 137 |
| 16 | *Havering* | 101204 | 95200 | 30 | 150 |
| 17 | *Hillingdon* | 107953 | 97071 | 33 | 161 |
| 18 | *Hounslow* | 98414 | 83224 | 29 | 142 |
| 19 | *Islington* | 100997 | 87886 | 23 | 123 |
| 20 | *Kensington and Chelsea* | 88317 | 70784 | 21 | 103 |
| 21 | *Kingston upon Thames* | 65637 | 59288 | 20 | 98 |
| 22 | *Lambeth* | 135932 | 119887 | 36 | 178 |
| 23 | *Lewisham* | 121254 | 108984 | 39 | 169 |
| 24 | *Merton* | 82394 | 75957 | 25 | 124 |
| 25 | *Newham* | 107582 | 92862 | 37 | 164 |
| 26 | *Redbridge* | 101564 | 93657 | 36 | 161 |
| 27 | *Richmond upon Thames* | 82841 | 77638 | 23 | 115 |
| 28 | *Southwark* | 129264 | 102348 | 34 | 166 |
| 29 | *Sutton* | 80945 | 71314 | 25 | 121 |
| 30 | *Tower Hamlets* | 112518 | 76988 | 33 | 144 |
| 31 | *Waltham Forest* | 99015 | 92416 | 28 | 144 |
| 32 | *Wandsworth* | 137275 | 119465 | 37 | 179 |
| 33 | *Westminster* | 122209 | 89339 | 24 | 128 |

**Supplementary Table 2.** (a) Demographics of Twitter users; and (b) Rate of Twitter usage among minority groups (i.e., sexual identity and disability status) in England, 2014-5.

**(a)**

| | |
|---|---|
| Male | 53.3% |
| Female | 46.7% |
| 16-24 | 15% |
| 25-44 | >30% |
| 45-64 | >30% |
| 65-74 | >10% |
| +75 | 10% |
| White | 86.8% |
| Non-white | 13.2% |
| Upper socioeconomic group | 57.4% |
| Lower socioeconomic group | 21.6% |

**(b)**

| | |
|---|---|
| LGB | 44.5% |
| Heterosexual/straight | 21.2% |
| Longstanding illness, disability or infirmity | 14.0% |
| No longstanding illness, disability or infirmity | 24.8% |

**Supplementary Table 3.** Spatial autocorrelation–Energy consumption and Human Mobility, Greater London, 2014.

| | *Moran's I* | | | *Geary's C* | | |
|---|---|---|---|---|---|---|
| | *I* | *p-value* | *STD* | *C* | *p-value* | *STD* |
| Energy | | | | | | |
| *Electricity* | 4.53E-01 | < 2.2e-16 | 53.256 | 0.560347 | < 2.2e-16 | 37.361 |
| *Gas* | 5.67E-01 | < 2.2e-16 | 66.617 | 0.429042 | < 2.2e-16 | 57.799 |
| Human Mobility | | | | | | |
| *December* | 0.308308 | < 2.2e-16 | 37.154 | 0.746873 | 3.28E-12 | 6.8667 |
| *January* | 2.69E-01 | < 2.2e-16 | 32.076 | 0.693414 | < 2.2e-16 | 10.367 |
| *February* | 0.164037 | < 2.2e-16 | 19.311 | 0.84659 | < 2.2e-16 | 12.719 |
| *March* | 1.72E-01 | < 2.2e-16 | 20.3 | 0.832689 | < 2.2e-16 | 12.799 |
| *April* | 1.84E-01 | < 2.2e-16 | 21.717 | 0.806821 | < 2.2e-16 | 14.556 |
| *May* | 1.83E-01 | < 2.2e-16 | 21.525 | 0.807257 | < 2.2e-16 | 13.303 |
| *June* | 1.03E-01 | < 2.2e-16 | 12.173 | 0.897269 | 2.17E-14 | 7.5501 |
| *July* | 2.03E-01 | < 2.2e-16 | 23.991 | 0.797352 | < 2.2e-16 | 11.652 |
| *August* | 3.79E-01 | < 2.2e-16 | 45.485 | 0.664461 | < 2.2e-16 | 9.9 |
| *September* | 2.82E-01 | < 2.2e-16 | 33.681 | 0.731413 | < 2.2e-16 | 9.3013 |
| *October* | 1.92E-01 | < 2.2e-16 | 22.64 | 0.800093 | < 2.2e-16 | 14.687 |
| *November* | 1.79E-01 | < 2.2e-16 | 21.061 | 0.815603 | < 2.2e-16 | 15.444 |

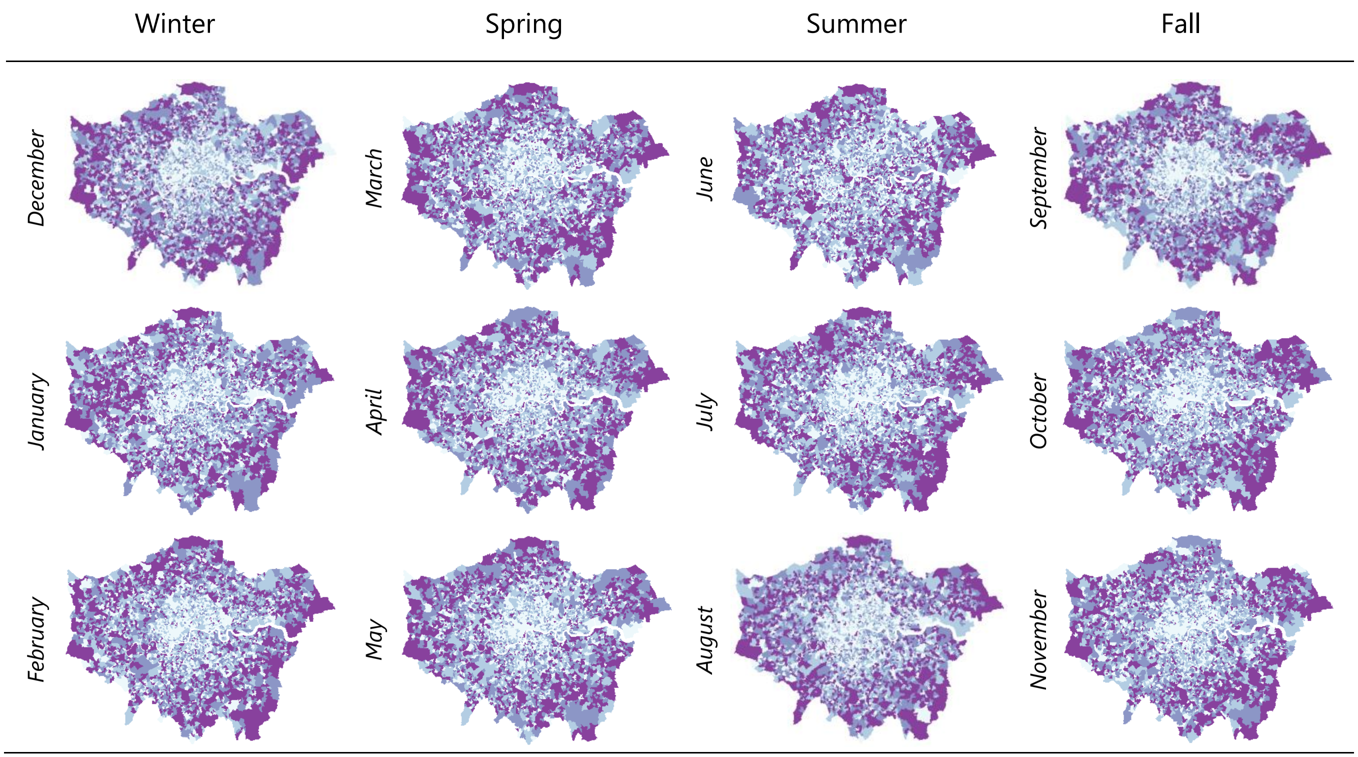


**Supplementary Figure 4.** Spatial distribution by month (2014): Human mobility.

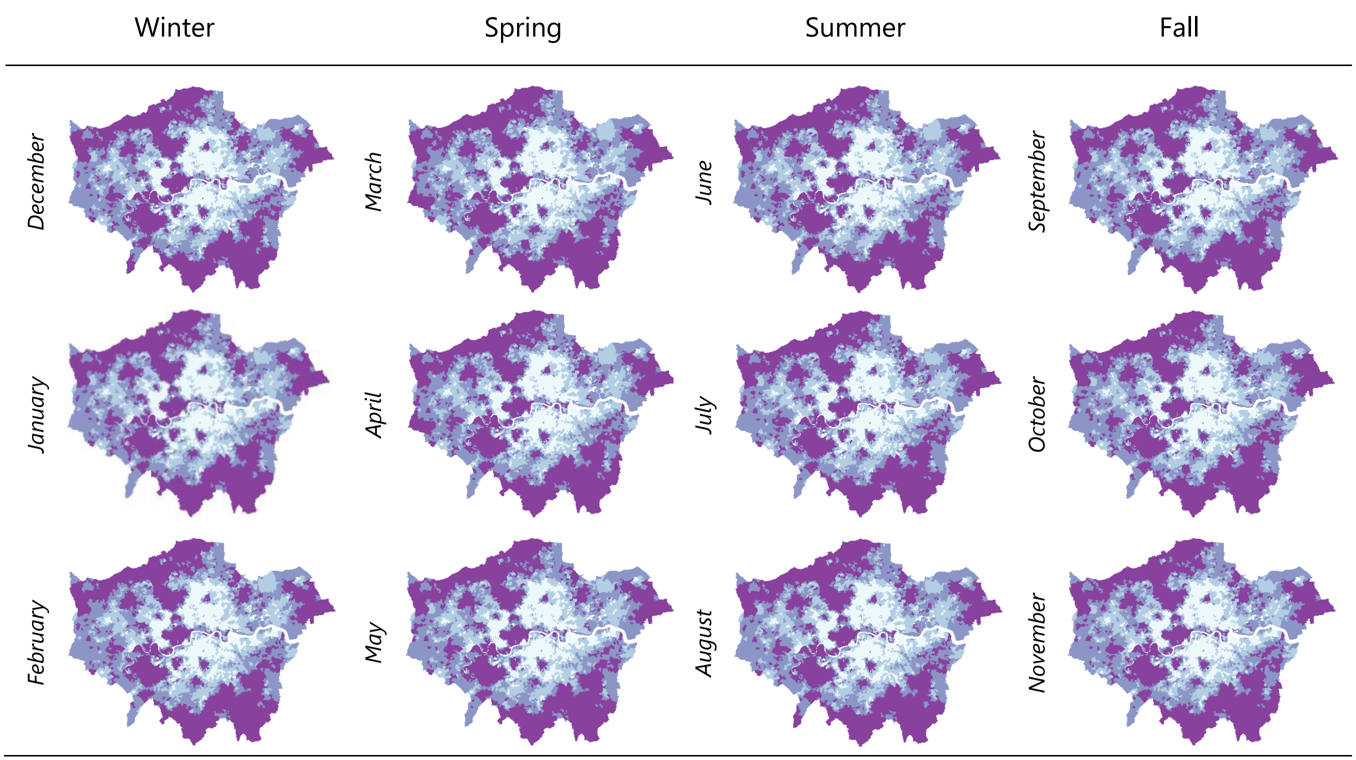


**Supplementary Figure 5.** Fitted SAR models for electricity consumption by month (2014), Greater London.

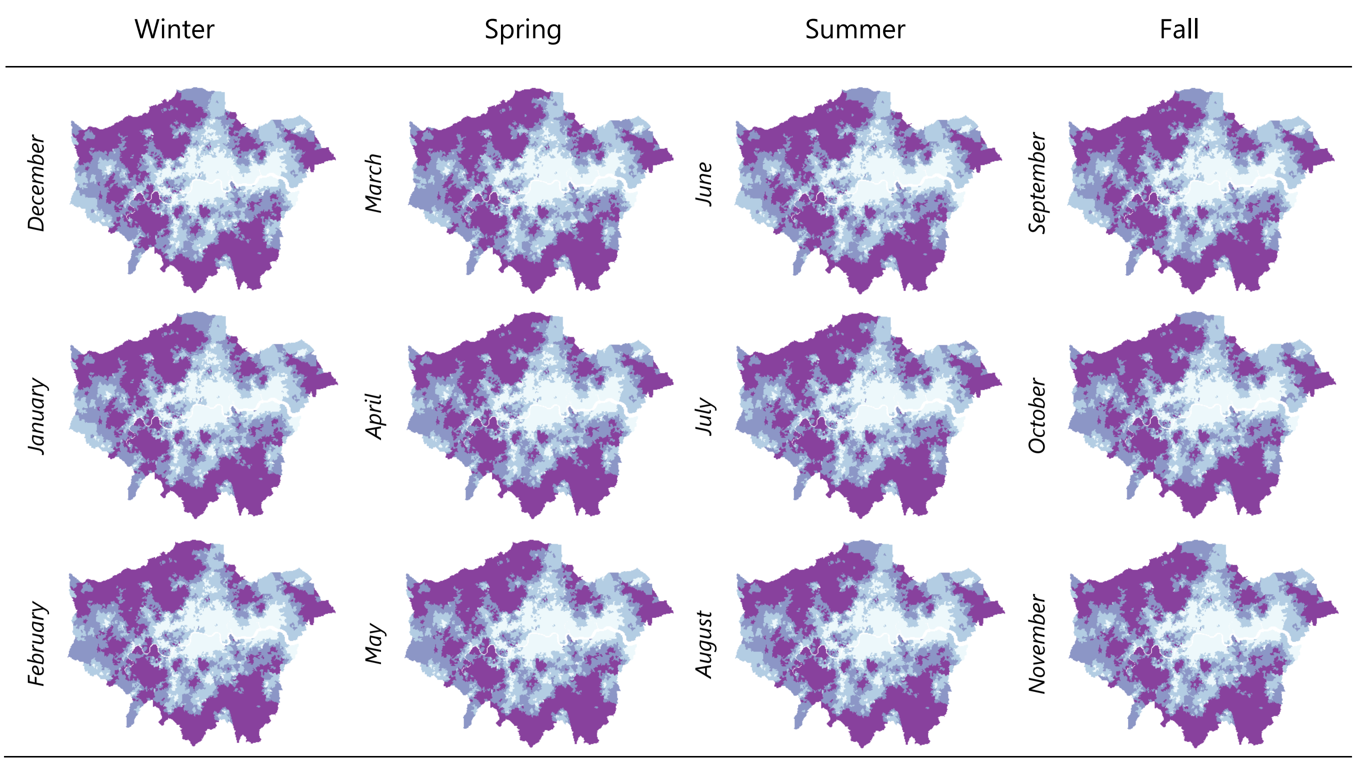


**Supplementary Figure 6.** Fitted SAR models for gas consumption by month (2014), Greater London.

**Supplementary Table 4.** Direct and indirect (spillover) effects of recurrent mobility on building energy consumption, Greater London, May 2014.

| | Moran's I | | | Spatial Durbin Model (SDM) | | | | | | | |
|---|---|---|---|---|---|---|---|---|---|---|---|
| | *I* | *p-value* | *STD* | *Rho* | *p-value* | *z-value* | *LL* | *AIC*[1] | *Direct* | *Indirect* | *Total* |
| *Commercial Electricity* | | | | | | | | | | | |
| 1 | 0.880276053 | < 2.2e-16 | 15.195 | 0.22953* | 0.079054 | 1.7562 | -152.8839 | 319.77 (320.34) | -0.07917506 | 0.0211709 | -0.05800416 |
| 2 | 0.940396814 | < 2.2e-16 | 16.051 | 0.3749*** | 0.00086107 | 3.3324 | -47.18423 | 108.37 (116.92) | 0.0012994 | 0.09462842 | 0.09592782 |
| 3 | 0.9367481966 | < 2.2e-16 | 39.658 | 0.52416*** | < 2.22e-16 | 12.437 | -52.45835 | 118.92 (247.79) | -0.004325174 | 0.11353599 | 0.1092108 |
| 4 | 0.70729262 | 6.417e-13 | 7.0961 | -0.15594 | 0.45354 | -0.74953 | -63.7796 | 141.56 (140.01) | 0.04449983 | -0.08192587 | -0.03742604 |
| *Residential Electricity* | | | | | | | | | | | |
| 1 | 0.863020134 | < 2.2e-16 | 21.598 | 0.60301*** | < 2.22e-16 | 9.7523 | -208.2073 | 430.41 (503.43) | 0.07146984 | 0.14785940 | 0.2193292 |
| 2 | 0.889785556 | < 2.2e-16 | 20.81 | 0.61089*** | < 2.22e-16 | 9.1298 | -146.409 | 306.82 (370.01) | -0.08958094 | -0.2093852 | -0.2989662 |
| 3 | 0.711790552 | < 2.2e-16 | 8.439 | 0.33982** | 0.024246 | 2.2532 | -22.76668 | 59.533 (61.331) | -0.02529448 | 0.2739436 | 0.2486491 |
| 4 | 0.956445763 | < 2.2e-16 | 18.843 | 0.42108*** | 1.8572e-05 | 4.2814 | -92.61926 | 199.24 (214.33) | -0.05521437 | 0.12596493 | 0.07075056 |
| 5 | 0.893790046 | < 2.2e-16 | 21.286 | 0.44902*** | 7.3183e-09 | 5.7835 | -158.691 | 331.38 (355.73) | 0.06296736 | 0.15663674 | 0.2196041 |
| 6 | 0.62594384 | 1.83e-08 | 5.5065 | 0.4352** | 0.019288 | 2.3399 | -21.15141 | 56.303 (57.01) | 0.07014332 | -0.1349026 | -0.06475931 |
| 7 | 0.562725350 | 5.148e-10 | 6.1047 | 0.36511** | 0.041991 | 2.0336 | -22.91907 | 59.838 (60.69) | 0.08862401 | 0.3660653 | 0.4546893 |
| *Commercial Gas* | | | | | | | | | | | |
| 1 | 0.9628519227 | < 2.2e-16 | 48.61 | 0.14636*** | 0.0041022 | 2.8702 | -1314.349 | 2642.7 (2649) | 0.02769916 | -0.03917283 | -0.011473671 |
| 2 | 0.580791230 | 4.035e-10 | 6.1436 | -0.43407** | 0.020875 | -2.3102 | 20.92904 | -27.858 (25.069) | -0.001018955 | -0.003396056 | -0.004415012 |
| *Residential Gas* | | | | | | | | | | | |
| 1 | 0.686805662 | < 2.2e-16 | 11.342 | 0.41145*** | 0.00050996 | 3.4755 | -102.9129 | 219.83 (228.07) | -0.001018955 | -0.003396056 | -0.004415012 |
| 2 | 0.739193250 | < 2.2e-16 | 8.8516 | 0.34964*** | 0.027013 | 2.2113 | -52.63074 | 119.26 (122.37) | -0.03221395 | 0.0401297 | 0.00791575 |
| 3 | 0.74570915 | 1.423e-13 | 7.3014 | 0.30159* | 0.091022 | 1.69 | -49.4754 | 112.95 (113.3) | 0.3758624 | -0.2006688 | 0.1751936 |
| 4 | 0.930995489 | < 2.2e-16 | 27.159 | 0.70825*** | < 2.22e-16 | 15.527 | -236.2426 | 486.49 (631.06) | 0.09532447 | 0.75647283 | 0.8517973 |
| 5 | 0.937871220 | < 2.2e-16 | 16.579 | 0.69912*** | < 2.22e-16 | 9.229 | -81.61937 | 177.24 (228.16) | 0.006568156 | 0.1343723 | 0.1409404 |
| 6 | 0.934156927 | < 2.2e-16 | 25.733 | 0.65673*** | < 2.22e-16 | 12.435 | -293.1482 | 600.3 (697.36) | 0.06833037 | 0.3199987 | 0.3883291 |
| 7 | 0.68855987 | 1.329e-09 | 5.9515 | 0.54555*** | 0.00047597 | 3.4939 | -41.53514 | 97.07 (101.34) | 0.19224910 | -0.5584959 | -0.3662468 |

*p<0.1*; p<0.05**; p<0.001****

[1]*AIC for ordinary least squares (OLS) models in parentheses.*
